# Spatial transcriptomic profiling to identify mesoderm progenitors with precision genomic screening and functional confirmation

**DOI:** 10.1111/cpr.13298

**Published:** 2022-07-30

**Authors:** Guanghui Liu, Guanheng Yang, Guijun Zhao, Chuanliang Guo, Yitao Zeng, Yan Xue, Fanyi Zeng

**Affiliations:** ^1^ Shanghai Institute of Medical Genetics, Shanghai Children's Hospital Shanghai Jiao Tong University School of Medicine Shanghai China; ^2^ Department of Histo‐Embryology, Genetics and Developmental Biology Shanghai Jiao Tong University School of Medicine Shanghai China; ^3^ NHC Key Laboratory of Medical Embryogenesis and Developmental Molecular Biology, Shanghai Key Laboratory of Embryo and Reproduction Engineering Shanghai China; ^4^ School of Pharmacy Macau University of Science and Technology Macau China

## Abstract

**Objectives:**

Mesoderm, derived from a new layer between epiblast and hypoblast during gastrulation, can differentiate into various tissues, including muscles, bones, kidneys, blood, and the urogenital system. However, systematic elucidation of mesoderm characteristics and specific markers remains a challenge. This study aims to screen and identify candidate genes important for mesoderm development.

**Materials and Methods:**

Cells originating from the three germ layers were obtained by laser capture microdissection, followed by microcellular RNA sequencing. Mesoderm‐specific differentially expressed genes (DEGs) were identified by using a combination of three bioinformatics pipelines. Candidate mesoderm‐specific genes expression were verified by real‐time quantitative polymerase chain reaction analysis and immunohistochemistry. Functional analyses were verified by ESCs‐EBs differentiation and colony‐forming units (CFUs) assay.

**Results:**

A total of 1962 differentially expressed mesoderm genes were found, out of which 50 were candidate mesoderm‐specific DEGs which mainly participate in somite development, formation of the primary germ layer, segmentation, mesoderm development, and pattern specification process by GO analysis. Representative genes *Cdh2*, *Cdh11*, *Jag1*, *T*, *Fn‐1*, and *Pcdh7* were specifically expressed in mesoderm among the three germ layers. *Pcdh7* as membrane‐associated gene has hematopoietic‐relevant functions identified by ESCs‐EBs differentiation and CFUs assay.

**Conclusions:**

Spatial transcriptomic profiling with multi‐method analysis and confirmation revealed candidate mesoderm progenitors. This approach appears to be efficient and reliable and can be extended to screen and validate candidate genes in various cellular systems.

## Graphical abstract

A schematic diagram to illustrate the experimental system for spatial transcriptomic analysis of late streak stage embryo and candidate gene screening. In summary, the transcriptomic profiling was obtained by RNA‐seq post‐laser capture microdissection of specific embryo regions. Fifty mesoderm‐specific differentially expressed genes (DEGs) were identified by using a combination of three bioinformatics pipelines. Candidate mesoderm‐specific genes expression was verified by real‐time quantitative polymerase chain reaction analysis and immunohistochemistry. Further functional analysis was performed by ESCs‐EBs differentiation. One candidate mesoderm‐related membrane‐associated gene Pcdh7 that thought to have hematopoietic‐relevant functions was identified as one of the mesoderm progenitor markers for tracking lineage cells in the late‐stage and was explored further with a CFUs assay. This approach appears to be efficient and reliable and can be extended to screen and validate candidate genes in various cellular systems.

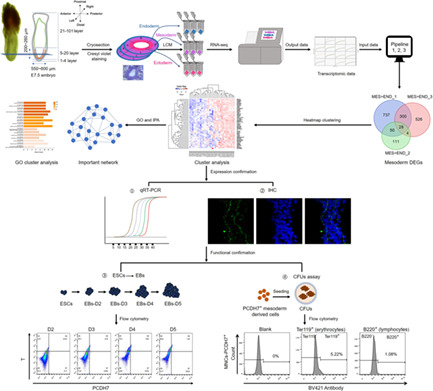

## 1 INTRODUCTION

Development from a fertilized egg to a gastrulating embryo requires precise regulation in a series of lineage‐specific and axis‐patterning events during mammalian embryogenesis. Nascent mesoderm is formed when epiblast cells ingress at the primitive streak (PS) undergoing epithelial‐mesenchymal transition.^1^, ^2^ Mesoderm is the middle of three germ layers first discovered and defined by anatomical investigations.^3^, ^4^ In the mouse embryo, a vital development feature is the formation of mesoderm starting around E6.5 during gastrulation, as cells migrate laterally from the PS into the spaces between ectoderm and visceral endoderm to form the middle mesoderm layer.^5^, ^6^, ^7^ In about 1 day, at E7.5, mesoderm cells expand into all sides of the embryonic region,^7^ and dramatically produce extraembryonic mesoderm, lateral mesoderm, paraxial mesoderm, gut, and notochord.^8^ Mesoderm cells have pluripotent ability to give rise to various tissues, including blood, bone, muscle, heart, vasculature, kidney, gonad, cartilage, and dermis.

Many gene expression regulatory systems and complex signaling interactions are critically involved in mesoderm induction during mouse gastrulation.^9^ Genetic studies show that the key signaling pathways include Wnt, transforming growth factor‐β (TGFβ), BMP4, Nodal, and fibroblast growth factor;^10^, ^11^, ^12^, ^13^, ^14^ and transcription factors, such as Brachyury (T), SOX2, OCT4, SMAD1, and SOX17, govern cell lineages from the three germ layers in mouse embryos.^15^, ^16^, ^17^, ^18^, ^19^ Nevertheless, the spatial and temporal regulation of genes in the developing embryos that direct specific cells toward the mesoderm fate is a complicated process. The molecular mechanism of how cells exit from the multipotent state, that is, cell lineage specification or the regionalization of cell fates during mouse embryo development, is still not fully known. The original method of identifying mesoderm in vertebrates was through anatomical and histological manipulations.^8^, ^9^, ^20^ How to precisely isolate mesoderm cells and quickly identify functions mesoderm‐specific genes is a great challenge. More methods are needed to identify a collection of mesoderm cells for mechanistic study and regenerative medicine applications.

Comprehensive characterization of the transcriptome during gastrulation is essential for understanding the molecular mechanism for lineage specification and embryonic patterning. Gene expression profiles of preimplantation or post‐implantation embryos have been studied by microarray analysis,^21^, ^22^ single‐cell quantitative polymerase chain reaction (qPCR),^23^ and single‐cell RNA sequencing (scRNA‐seq) analysis,^18^, ^24^, ^25^ and more. Some recent studies have reported mesoderm‐specific genes in mouse embryos at the late streak stage (at E7.5) by microcellular RNA‐seq.^26^, ^27^ However, how comprehensive the candidate gene lists are remains a question, as most of the candidate genes were not verified, and some of the previous well‐known genes such as T were not included in some of the reported gene lists.^27^

The identification of mesoderm‐specific genes would benefit from high‐throughput bioinformatics analysis. The choice of bioinformatics analytical methods can sometimes result in inconsistent lists of differentially expressed genes (DEGs) for further functional confirmation studies. In one study,^28^ authors reported the use of several different mapping methods to treat two independent RNA‐seq datasets, and the algorithms, parameters, and statistical models used in these different software/tools of the analysis pipeline appeared to bring bias for final summary DEGs. Thus, a more reliable bioinformatics analysis would be useful.

Genetic methods using mouse models have been applied to identify functions of genes from observation of phenotypes in mouse embryo development.^29^, ^30^, ^31^, ^32^ Other methods such as in situ hybridization, β‐galactosidase staining, or GFP tracing were used to identify the expression pattern of genes (e.g., *T*, *Lhx1*, *Flk‐1*, and *Eome*) in different stages of the embryo.^19^, ^33^, ^34^, ^35^ These strategies usually are low through‐put, often time‐consuming, and the known marker genes might not be specifically expressed in the limited mesoderm during gastrulation; some genes could also be functioning in ectoderm or endoderm in different embryo development stages.^36^, ^37^, ^38^ Alternatively, mesoderm‐like cells could be induced during embryonic stem cell (ESCs) differentiation into embryoid body (EB).^39^ This in vitro differentiation system can be helpful to mimic the embryonic development stages during the three germ layers formation.

It is known that there is extensive crosstalk between the three germ layers during gastrulation,^26^ and spatial transcriptome analysis of cell populations in the three germ layers at a single‐cell resolution could add much information to our current understanding of the biological processes and functions of the different germ layers. While single marker labeled mesoderm cells were shown to have distinct cell fate,^40^ multi‐marker methods could also be used to illustrate cell fates of subgroup mesoderm cells located in specific locations at a certain developmental time point. The identification of mesoderm‐related genes is of particular interest; not only can they be used to trace the specific cell lineages so to predict cell fates, but also to study functional roles in regulating cell development and differentiation.

Thus, in this study, we documented the regional spatial transcriptomic profiling of the ectoderm, mesoderm, and endoderm, particularly at the mesoderm‐enriched embryo cross‐sections, in late‐streak‐stage E7.5 embryos, a stage that has previously been considered an ideal time point for mesoderm cell studies.^2^, ^41^ Using a combination of bioinformatics strategies, candidate mesoderm‐specific genes were identified and confirmed at transcription and/or translational levels. A rapid functional confirmation assay was performed and one novel candidate gene was used as a specific tracer to isolate a sub‐population of mesoderm‐derived cells. Together, we describe here a simple, efficient, and reliable system to verify specific mesoderm genes.

## 2 MATERIALS AND METHODS

### 2.1 Mice

A total of 129 mice were purchased from Shanghai Nan Fang Model Biotechnology, Ltd. (Shanghai, China) and kept in the specific pathogen‐free (SPF) animal facility of the Shanghai Institute of Medical Genetics, Shanghai Children's Hospital. Relevant animal experiments were reviewed and approved by the Laboratory Animal Management and Ethics Committee of Shanghai Children's Hospital (review opinion number LLSC2016019). All animal experiment operations met the ethical requirements of the committee and were conducted under supervision.

### 2.2 Embryo hematoxylin and eosin (H&E) staining, laser capture microdissection (LCM)

Surgical procedures were performed in compliance with protocols approved by the Laboratory Animal Management and Ethics Committee of Shanghai Children's Hospital. Timed mating was set up between 129 mice. E7.5 embryos^20^ of 129 mice were embedded in optimum cutting temperature compound (Sakura) and cryosectioned serially at 16‐μm thick. For H&E staining, frozen sections were soaked in water for 1 min and then stained in hematoxylin (Yeasen) for 4 min. The slides were stained in eosin (Yeasen) for 20 s after water immersion for 5 min. Then, the slides were dehydrated in a series of 70%, 95%, 95%, 100%, and 100% ethanol (Chemisci) (1 min for each step). Before being sealed with neutral resins, the slides were immersed in xylene (Chemisci) two times (5 min each time). For LCM, frozen sections were allowed to quickly thaw at room temperature (RT) and then dehydrated in ice‐cold 100% ethanol for 30 s. Fixation was performed in 95% ethanol for 60 s. The slides of intraembryonic coelom were stained for 60 s with 1% cresyl violet acetate solution (Sigma‐Aldrich, prepared in 75% ethanol), dehydrated in a series of 95%, 95%, 100%, 100%, 100% ethanol (30 s for each step), and finally subjected to LCM on a PALM laser microdissection (Carl Zeiss). Approximately, 50–300 cells from one germ layer in each section were harvested by LCM. The LCM was done as quickly as possible to protect RNA integrity in samples.

### 2.3 RNA isolation and microcellular RNA‐seq

Cell samples post‐LCM were treated by a modified Smart‐seq2 protocol for LCM samples.^42^ Briefly, cell samples were lysed in 50 μl of 4 M guanidine isothiocyanate solution (Invitrogen) at 42°C for 10 min. The volume of the lysate was adjusted to 200 μl by nuclease‐free water and was further concentrated by ethanol precipitation in the presence of 1/10 volume of acetate sodium (pH 5.7, 3 M) (Ambion) and 2 μl of carrier glycogen (20 mg/ml) (Roche). Total RNA pellets were dissolved in lysis solution and used as a template for following procedures.

cDNA Libraries were prepared using the Illumina Nextera XT DNA preparation kit (Illumina), and pooled libraries of 96 cells were sequenced on the Illumina Hi‐Seq 2500 (Illumina).

### 2.4 Bioinformatics analysis for the identification of DEGs in mesoderm

FastQC was used to evaluate raw sequence data.^43^ Three analytical pipelines were designed for the bioinformatics analysis to identify mesoderm‐related DEGs. STAR‐HTseq‐count‐DESeq2 (Pipeline 1), HISAT2‐StringTie‐Ballgown (Pipeline 2), and HISAT2‐SummarizeOverlaps‐DESeq2 (Pipeline 3) were implemented for the analysis of mesoderm‐specific DEGs independently. The three analytical pipelines were operating on algorithms utilizing different open‐source software tools.^44^, ^45^, ^46^, ^47^, ^48^ For Pipeline 1, STAR (version 2.4.1a)^45^ was used to map reads Mus musculus genome (Ensembl version 38.91), HTseq‐count was used to count reads for calculating the gene expression levels (default options), DESeq2^46^ was used to find DEGs. For Pipeline 2 and 3, the three functions were carried out by HISAT2 (version 2.1.0)‐StringTie‐Ballgown^48^ and HISAT2 (version 2.1.0)‐SummarizeOverlaps‐DESeq2^47^ to find DEGs, respectively. The condition of *p* < 0.01 and log2FoldChange >0 were used to find DEGs by the above three pipelines. For mesoderm‐specific DEGs, the mesoderm data were compared with ectoderm or endoderm independently, using the three pipelines, and finally union and intersects of the DEGs were processed.

GO enrichment analysis of DEGs was performed with the R package clusterProfiler (version 3.14) to identify enriched biological processes pathways.^49^ Our resulting DEGs as well as two candidate gene lists from the public database^26^, ^27^ were subject to GO enrichment analysis.

Pathway analysis was performed using QIAGEN Ingenuity Pathway Analysis (IPA, 2022, Qiagen).

### 2.5 Quantitative reverse transcription polymerase chain reaction (qRT‐PCR) analysis

cDNA Libraries used for microcellular RNA‐seq was also used as template in qRT‐PCR confirmation assay using FastStart Universal SYBR Green Master (Roche). The qRT‐PCR was performed with the LightCycler 96 (Roche) system.

Sequences of forward and reverse primers used in qRT‐PCR analysis were as follows: *β‐actin* forward primer: 5′‐CATCCGTAAAGACCTCTATGCCAAC‐3′, *β‐actin* reverse primer: 5′‐ATGGAGCCACCGATCCACA‐3′; *Cdh2* forward primer: 5′‐AGCGCAGTCTTACCGAAGG‐3′, *Cdh2* reverse primer: 5′‐TCGCTGCTTTCATACTGAACTTT‐3′; *Cdh11* forward primer: 5′‐CTGGGTCTGGAACCAATTCTTT‐3′, *Cdh11* reverse primer: 5′‐GCCTGAGC CATCAGTGTGTA‐3′; *Jag1* forward primer: 5′TGTGTGAAGTTGGAAGCATCC‐3′, *Jag1* reverse primer: 5′‐ACCTTGAGCTTGGTAATAGCAC‐3′; *Pcdh7* forward primer: 5′‐ CAGCCATTTCGTAGAGTGACG‐3′, *Pcdh7* reverse primer: 5′‐ CTTGGTGTTTCTGACTCCTCC‐3′. GraphPad Prism 8 software was applied to analyze the qRT‐PCR data. The data were evaluated by Kolmogorov–Smirnov test. *p* value <0.05 indicated that the results were statistically significant. *p* Value <0.01 indicated that the results were statistically extremely significant.

### 2.6 IHC and confocal image analysis

Cryosections of the middle session of the E7.5 intraembryonic region were fixed in iced acetone at −20°C for 10 min, blocked with goat serum at RT for 1 h, and then incubated with primary antibodies, diluted in PBS + 0.2% BSA + 0.1% TritonX‐100, at 4°C overnight. The cryosections were incubated with Alexa Fluor secondary antibodies (Invitrogen) for 1 h at RT, then incubated with DAPI for 5 min at RT. After the second round of fixation, cryosections were ready for imaging. Leica DMRXA2 was used to collect immunofluorescence images. The primary antibodies used in our study are listed: FN1 (abcam), CDH2 (abcam), and T (abcam).

### 2.7 Cell culture and EB differentiation assay

Mouse embryonic stem cell line CGR8.8 cells (generously provided by Dr Yanru Chen from Stanford University) were cultured in an ESCs culture medium^50^ that consists of 15% FCS (PAA), 1x non‐essential amino acid (Gibco), 1% Pen/Strep (Gibco), 4 mM L‐glutamine (Gibco), 0.1 mM β‐mercaptoethanol (Gibco), 1000 U/ml LIF (Millipore), and DMEM (Gibco).

EB formation was used as a quick functional assay to predict the cell fate of the three germ layers in vitro. CGR8.8 cells were allowed to differentiate when cultured in ESCs culture medium without LIF. EB morphology was observed on Day 2 through Day 5. EBs were treated with 0.25% trypsin (BasalMedia) to dissociate individual cells and then subjected to flow cytometry analysis.

### 2.8 Flow cytometry analysis

Single‐cell suspension at a density of 1 × 10^*5*^/100 μl after EB 0.25% trypsin digestion treatment was incubated with antibodies at 4°C for 30 min in 100 μl of PBS. Flow cytometry experiments were performed with Beckman Coulter CytoFLEX S (Beckman) and analyzed using FlowJo software (Tree Star). Antibodies used were the following: anti‐mouse FLK‐1 (BD Pharmingen), anti‐mouse CDH2 (abcam), anti‐mouse CDH11 (Invitrogen), anti‐mouse JAG1 (eBioscience), anti‐mouse PCDH7 (biorbyt).

### 2.9 Colony‐forming units (CFUs) assay for hematopoietic progenitor cells

Embryonic development time was estimated by considering the day of vaginal plug observation as E13.5 FL was dissected and isolated from E13.5 embryos. Furthermore, FL mononuclear cells (FL‐MNCs) were isolated by Ficoll separation using mouse Ficoll separation solution kit (TBD science).

First, PE‐anti‐PCDH7 was generated by PCDH7 antibody (biorbyt) and PE conjunction Kit (abcam). Subsequently, the cells were magnetically labeled with anti‐PE MicroBeads (Miltenyi Biotec, Inc.). Then, the cell suspension was loaded on a MACS Column which was placed in the magnetic field of a MACS Separator. The magnetically PCDH7 antibody‐labeled cells were retained in the column while the unlabeled cells ran through. After removal of the column from the magnetic field, the PCDH7^+^ cells could be eluted as the positively selected cell fraction.

In vitro hematopoietic progenitor analysis was performed on dilutions of sorted cells plated in triplicates in MethoCult media (StemCell Technologies) to form CFUs. Plates were incubated at 37°C in a humidified chamber under 5% CO_2_. Hematopoietic colonies were counted with an inverted microscope at Day 13 of the culture.

Flow cytometric analysis was performed on CytoFLEX (Beckman). Staining was performed in PBS/FCS for 30 min at 40°C. The positive gates were defined from staining with isotype‐matched control antibodies. BV421‐anti‐Ter119, BV421‐anti‐BB220, flow cytometry data were analyzed with CytExpert software.

## 3 RESULTS

### 3.1 Characteristics of whole‐mount late streak stage embryo

**FIGURE 1:**
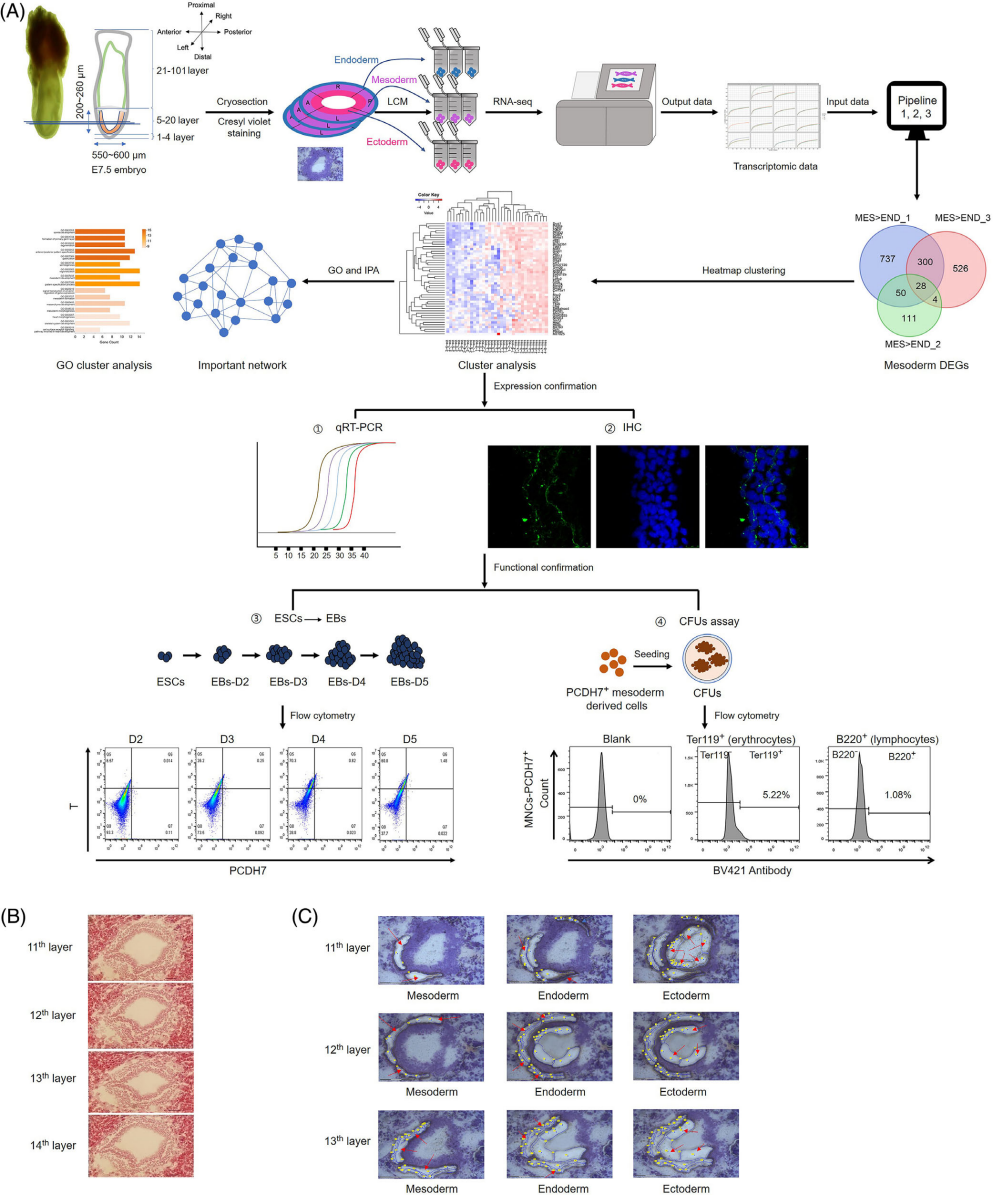
Spatial transcriptomic profiling to screen mesoderm DEGs and identify mesoderm markers with functional confirmation. (A) A schematic diagram to illustrate the experimental system for spatial transcriptomic analysis of late streak stage embryo and candidate gene screening and confirmation. Top left, a sketch of representative E7.5 embryo that fits the selection criteria. (B) H&E staining of the cross‐sections located at the middle positions that have enriched mesoderm cells from a late streak stage embryo (E7.5). The number of the embryo cryosections layer is shown. (C) LCM‐captured images of three germ layers located in the middle of the intraembryonic region from a representative E7.5 embryo. The positions marked with a red arrow correspond to the three germ layers. The yellow dots are LCM sample capture sites

A schematic diagram to illustrate the experimental system for spatial transcriptomic analysis of late streak stage embryo and candidate gene screening are shown in Figure 1A. E7.5 embryos were collected to visualize mesoderm structure according to morphological landmarks under the dissecting microscope^20^ (Figure 1A). The anterior end of the primitive streak condenses into the ‘node’ at the distal tip of the egg cylinder, and the posterior amniotic fold is observed, consistent with that of the late streak embryos.^20^ Whole‐mount late streak stage embryos (at E7.5) were cryosectioned with each sectioned layer being 16‐μm thick, followed by H&E staining to observe the morphological structure of the three germ layers.

For clear cell capture for spatial transcriptome analysis, the histological view of the cross‐section of the whole‐mount late streak stage embryo is used to guide the evaluation of feature of mesoderm at different embryo regions in the decidua enclosed embryos. In Figure 1B, four cross‐sections of the intraembryonic region of a representative E7.5 embryo are shown (Layers 11–14). From distal to posterior, the selected embryo (Figure 1A, top left) would typically have about 100 layers; the first 4 layers were the decidua region, and the following 16 layers were mainly in the intraembryonic region. The remaining four‐fifths of the embryos were in the extraembryonic coelom region. Since the size of the embryos increases dramatically and dynamically during gastrulation, for our experiments, in order to minimize the variable sub‐stages of the embryos, only embryos with the following features were selected for further studies, according to E7.5 embryos morphology characteristics described from previous studies.^7^, ^20^, ^51^ First, the height of the inner embryo is 200–260 μm; second, the maximum diameter of the intraembryonic region is 550–600 μm; third, a clear structure of three germ layers has to be visualized at the intraembryonic region sections.

To minimize the heterogeneity of the embryo samples, the middle sections of the embryonic coelom where there were most visible mesoderm cells were used for LCM (Figure 1C). In addition, the choice of 16 μm per layer thickness, as compared to 20 μm used in another study,^26^ was from our previous experience and estimation that each LCM mesoderm sample would have enough cells for the subsequent analyses while reducing data variability due to the heterogeneous nature of mesoderm cells. Furthermore, the use of LCM, which is currently the most precise cell capture method, compared to the previous method of dissecting with glass needles,^27^ would add one more layer of detailed and accurate positional information to the cells. Altogether, the samples used for the study were as carefully designed and prepared as possible.

### 3.2 Capture of cells from the three germ layers

LCM was an efficient tool to apply for the spatial transcriptomics analysis. Here, LCM was performed on the mesoderm‐enriched embryo cross‐sections of four whole‐mount late streak stage E7.5 embryos that fit the above criteria. According to their morphology, all three germ layers were captured separately on each cryosection^7^ (Figure 1C). Thirty‐four samples were prepared for LCM, followed by spatial transcriptome analysis using microcellular RNA‐seq.

The mesoderm cells in different positions have different cell fates, hence the heterogeneity and different sublayers of information in the mesoderm cells. Thus, in addition to the clarity of the three germ layer structures, the other reason to choose the middle of the intraembryonic region for the study is that they have the potential to differentiate into hematopoietic progenitor, endothelial progenitor, and cardiac mesoderm from previous studies^25^, ^40^ that have been a particular interest for us.

### 3.3 Screening of mesoderm candidate genes

The spatial transcriptomic data of the three germ layer samples were obtained by microcellular RNA‐seq. For data analysis, three data analytical pipelines (Pipeline 1, 2, 3) were designed to be used in combination to screen for candidate genes. In particular, after raw data quality control, the three pipelines used a mixture of some of the most popular software that has been considered fast and reliable by many researchers^48^, ^52^ to complete the three essential analytical steps of mapping, read counting, and differential analysis. Pipeline 1 used a well‐established set of programs based on STAR,^45^ HTSeq,^44^ and DESeq2^46^ software that relied on more computational power. Pipeline 2 was composed of three recently popular and reliable combinations of tools focusing on reducing computational burden of differential expression analysis of assembled transcriptomes, based on HISAT2, StringTie, and Ballgown.^48^ Pipeline 3 was specifically designed for our research system that is based on HISAT2 for mapping on reference genome, summarizeOverlaps^47^ for reads counting, and DESeq2 for mapping DEGs. On one hand, three pipelines could cast a broader net to include a more complete list when taking the compilation of the three candidate DEGs; and on the other hand, three pipelines have more confidence for reducing possible bias from a single analytical tool, when taking the intersect of the three DEGs sets. The three pipelines together will help maximize the accuracy and efficiency of the analysis.

**FIGURE 2:**
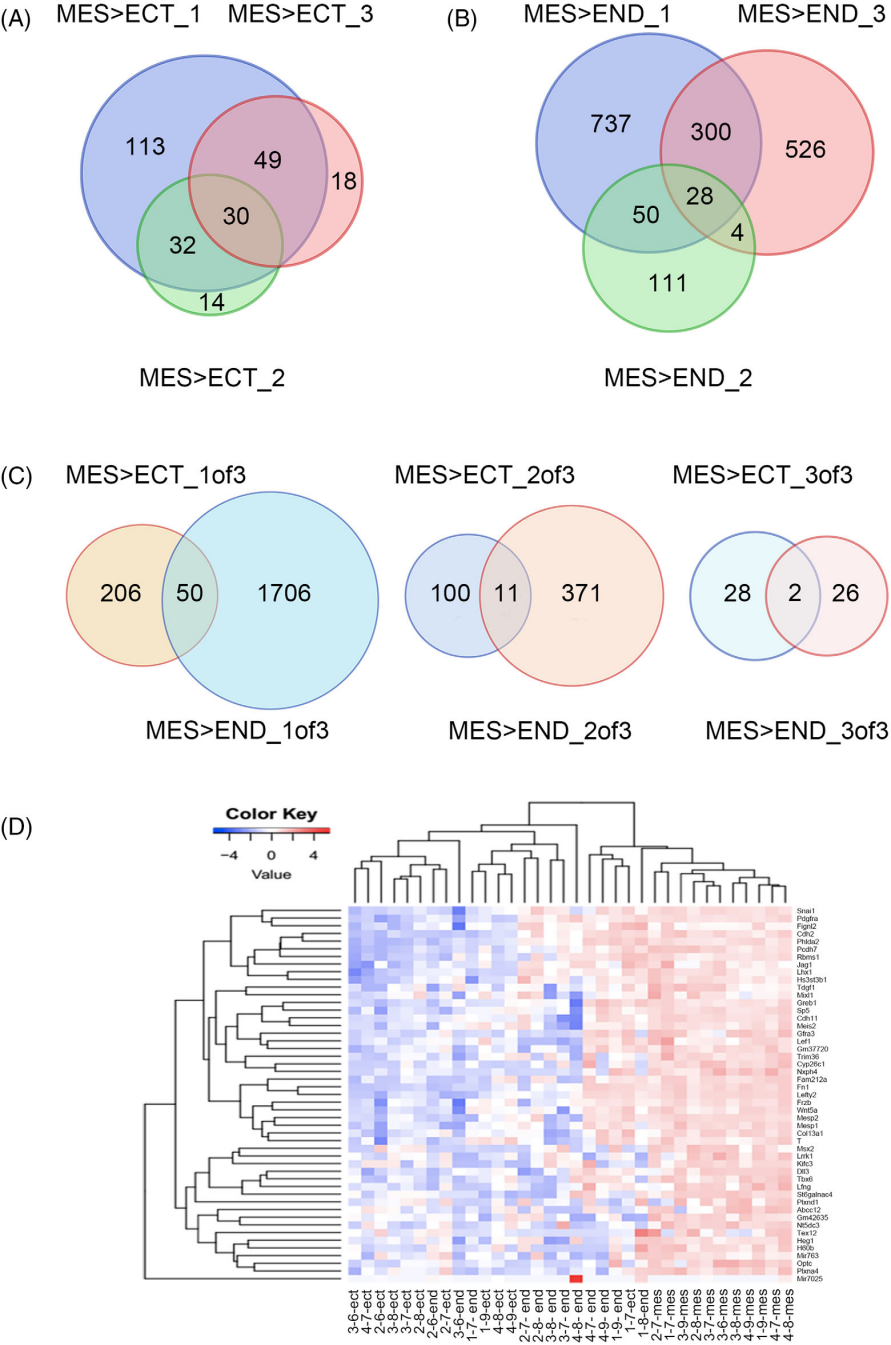
Screening of candidate mesoderm‐specific genes in the late‐gastrulation embryo. (A,B) The DEGs with significantly higher expression in mesoderm than either ectoderm and endoderm from overlap of all three pipeline analyses visualized through a Venn diagram. ECT, ectoderm; MES, mesoderm; 1, Pipeline 1; 2, Pipeline 2; 3, Pipeline 3. (C) Mesoderm‐specific DEGs visualized through Venn diagrams; left: from collection of at least one of three pipeline analyses; middle, from overlap of at least two out of three pipeline analyses; right, from overlap of all three pipeline analyses. (D) Heatmap of candidate mesoderm‐specific genes in three germ layers samples

The results revealed that 1962 genes have statistically significant higher expression in mesoderm compared to ectoderm or endoderm, among which 50 showed significantly higher expression in mesoderm than both of the ectoderm and endoderm layers (Figure 2A–C, left). The 50 DEGs showed higher expression in mesoderm than both the ectoderm and endoderm, boosting confidence in the system and indicating the stringency of the strategy used. Half of these genes had not been reported previously as functioning in early embryonic development, especially on site of gastrulation.

GO enrichment analysis with the 1962 candidate DEG sets showed that the prominent functions, such as multicellular organism development, animal organ morphogenesis, cell differentiation, axon guidance, and nervous system development, appeared to be of a broader and more general nature regarding embryogenesis (Table S3).

**FIGURE 3:**
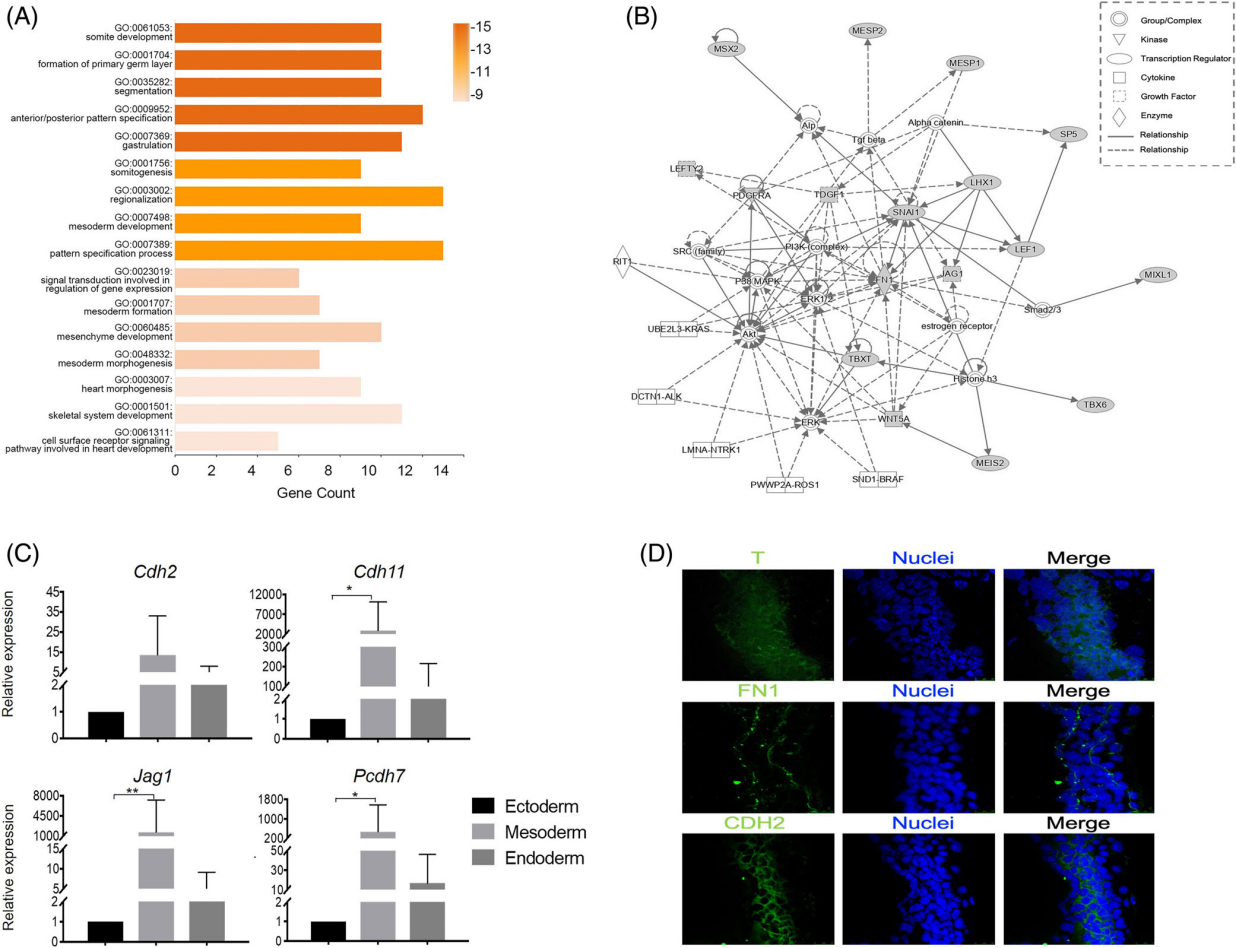
Network functions of candidate mesoderm‐specific genes by GO and IPA and their confirmation by qRT‐PCR and IHC. (A) GO cluster analysis of candidate mesoderm‐specific genes. (B) Top 1 network generated by IPA for the 50 mesoderm DEGs. The IPA network is graphically represented as nodes (proteins) and edges (the biological relationship between the nodes) and generated based on their functional and biological connectivity. The length of an edge reflects the evidence in the literature supporting that node‐to‐node relationship. Shaded notes represented mesoderm‐related genes screened by three analytical pipelines, while others (empty nodes) were not assessed in this study but identified by IPA as important nodes involved in the network. (C) qPCR expression analysis for selected mesoderm candidate genes (*Cdh2*, *Cdh11*, *Jag1*, and *Pcdh7*) in three germ layers. The mRNA expression of mesoderm candidate genes relative to β‐actin expression level. (D) IHC protein expression for selected candidate genes (*T*, *Fn1*, and *Cdh2*) in cross‐section of E7.5 embryo. Data are shown as mean ± SD. *p < 0.05, **p < 0.01

Clustering analysis of the 50 genes resulted in distinct cluster sets with mesoderm samples (Figure 2D). GO analysis of the 50 candidate mesoderm‐specific genes revealed that they participate in somite development, formation of the primary germ layer, segmentation, mesoderm development, and pattern specification process (Figure 3A, Table S2); most functions are relevant to the roles of mesoderm function in embryo development. IPA pathway analysis showed that the top network involving FN1, SNAI1, TDGF1, PDGFRA, JAG1, TBXT(T), and WNT5A formed a network functioning in embryo, organismal, and tissue development (Figure 3B). These functions are consistent with mesoderm biological process during gastrulation. Interestingly, some networks with 50 DEGs were related with blood system, such as hematological system development and function, hematological disease, and immunological disease (Table S4).

Thus, by a precise cell capture method LCM, together with a combination of three robust data analysis algorithms, we were able to generate a great number of candidate genes and, at the same time, a high‐confidence subset for specific functions such as membrane surface proteins. We also examined the collection of mesoderm‐specific DEG lists resulting from the overlap of at least two out of the three pipeline analyses, which yielded a 482 candidates gene list, among which 11 were differentially expressed in mesoderm compared to both ectoderm and endoderm (Figure 2C, middle). Ten out of the 11 genes were novel for mesoderm‐related functions (Table S1). Furthermore, using the most stringent condition requiring DEGs to overlap all three pipeline analyses, a total of 56 DEGs resulted from the union of 30 with significantly higher expression in mesoderm than ectoderm and 28 higher in mesoderm than endoderm. Two, *Fn1* and *Pcdh7*, intersect the two lists (Figure 2C, right). Interestingly, these two genes both encode membrane proteins (Table S1).

Surface membrane proteins are of particular interest because not only could they be very useful surface markers to isolate and trace candidate pluripotent stem cells, but they could also play essential roles in regulating pluripotency and differentiation.^53^ Indeed, the full gene set seems to be enriched for membrane protein‐associated genes (38%).

### 3.4 Identification and confirmation of mesoderm candidate genes

Different research teams have previously revealed mesoderm‐related candidate genes,^26^, ^27^ although confirmation of these genes remains warranted. In order to identify reliable candidate mesoderm‐specific genes and test the robustness of our system, we selected one classic mesoderm gene *T* and five membrane‐associated genes (*Cdh2*, *Cdh11*, *Jag1*, *Pcdh7*, and *Fn1*) from 50 mesoderm‐specific genes set for identification. The membrane‐associated or related proteins could be useful to trace, sort, and purify living cells for further studies.^53^

qRT‐PCR was used to confirm mesoderm‐associated genes at the transcription level. As shown in Figure 3C, the representative genes *Cdh2*, *Cdh11*, *Jag1*, and *Pcdh7* were expressed the highest in mesoderm among the three germ layers. The result is consistent with the RNA‐seq data (Figure S1).

IHC is another efficient method to visually reveal the location and abundance of the candidate proteins in three germ layers in situ. As a transcription factor, T has a role in the formation and organization of mesoderm and is regarded as a traditional mesoderm marker.^19^, ^54^, ^55^ Two other candidate genes, *Fn1* and *Cdh2*, which express at relatively high abundances and are located mainly at the membrane, were selected to further validate their location in three germ layers on embryo cryosections with limited cell numbers. As shown in Figure 3D, in the middle of the E7.5 intraembryonic region, T, was specifically expressed at the cell nucleus of the primitive streak cells; FN1 was shown to express on the membrane outlining the mesoderm that separated it from the ectoderm and the endoderm, and CDH2 is specifically expressed on the membrane of the mesoderm (Figure 3D). These results indicated that the samples captured from cryosections by LCM were located precisely in the mesoderm area as expected.

### 3.5 EB differentiation assay for mesoderm candidate genes validation

As a simple, functional confirmation assay, gene expression analysis of ESCs‐EBs differentiation (from Day 2 to Day 5) can be used to simulate the early embryonic development during mesoderm differentiation in vitro.^39^ This ESCs‐EBs differentiation system could provide enough cells, and the system is simpler and more time‐efficient than the genetically engineered mouse model for gene function studies. Four membrane‐associated candidate genes (*Cdh2*, *Cdh11*, *Jag1*, and *Pcdh7*) and two mesoderm marker genes (*T* and *Flk‐1*) were used to verify protein expression and molecular function during mesoderm differentiation in vitro. T is thought to be expressed in all nascent mesoderm, and can be used to track nascent mesoderm cells.^55^, ^56^ FLK‐1 is a membrane protein that can be used to track mesoderm cells that are essential for hemangiogenic lineage derivation,^31^, ^35^, ^57^ and it is mainly located in the intraembryonic region near the proximal and yolk sac. Thus, T and FLK‐1 can be used in combination to confirm the reliability of the ESCs‐EBs differentiation system that could mimic the generation of embryonic cells and extraembryonic cells. CDH2, CDH11, JAG1, and PCDH7 are also membrane proteins that are expressed and participate in the gastrulation embryo;^58^, ^59^, ^60^, ^61^, ^62^ however, their roles in mesoderm development during gastrulation remain unclear.

**FIGURE 4:**
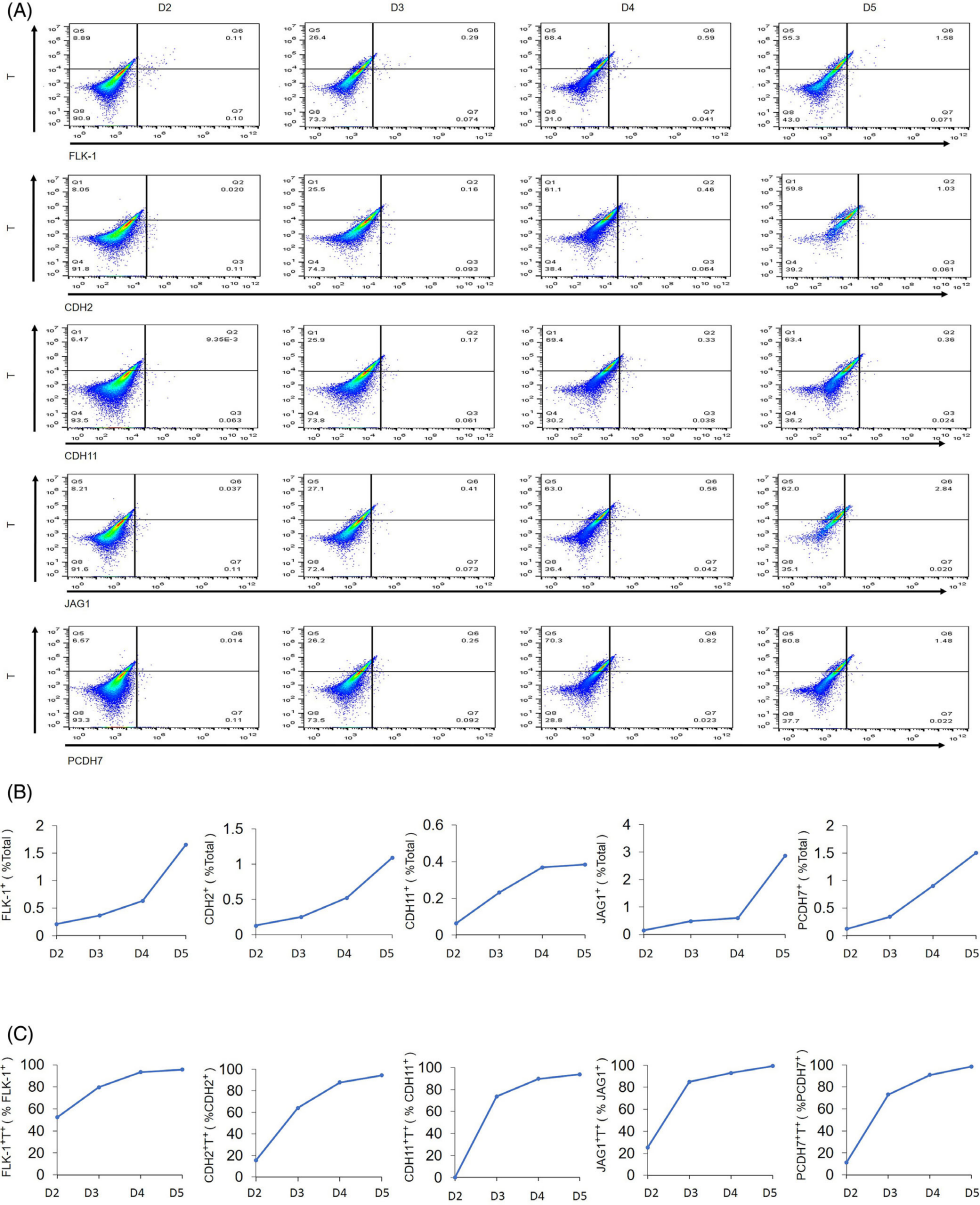
Identification of candidate mesoderm‐specific genes ESCs‐EBs differentiation. (A) Flow cytometry analysis of FLK‐1, CDH2, CDH11, JAG1, and PCDH7 combined with T during embryonic body differentiation from Day (D) 2 to Day 5. (B) Percentage of FLK‐1^+^, CDH2^+^, CDH11^+^, JAG1^+^, and PCDH7^+^ cells in embryonic bodies from Day 2 to Day 5. (c) Percentage of T^+^ cells in FLK‐1^+^, CDH2^+^, CDH11^+^, JAG1^+^, and PCDH7^+^ cells from Day 2 to Day 5

T has been considered one of the earliest markers of mesoderm development in ESCs studies and during gastrulation.^63^ Cells expressing T would be mesoderm‐like cells with mesoderm potential during ESCs‐EBs differentiation and they can be used as a positive control for mesoderm‐like cells in this in vitro differentiation system.^39^ Double screening of T and other DEGs would potentially be informative for studying subgroup of mesoderm cells. Co‐expressions of T with FLK‐1, CDH2, CDH11, JAG1, or PCDH7, during ESCs‐EBs differentiation were examined (Figure 4A,B). Results showed that FLK‐1^+^, CDH2^+^, CDH11^+^, JAG1^+^, and PCDH7^+^ cells increase during ESCs‐EBs differentiation from Day 2 to Day 5; the percentage of FLK‐1^+^, CDH2^+^, CDH11^+^, JAG1^+^, and PCDH7^+^ cells that are T^+^ cells increased accordingly, specifically, from over 60% by Day 3 to over 85% after Day 4 from EBs (Figure 4C). This result indicated that these FLK‐1^+^, CDH2^+^, CDH11^+^, JAG1^+^, and PCDH7^+^ cells are gradually differentiated into mesoderm‐like cells,^64^ which are T^+^FLK‐1^+^, T^+^CDH2^+^, T^+^CDH11^+^, T^+^JAG1^+^, and T^+^PCDH7^+^ cells during embryonic development. Altogether, these four candidate genes (*Cdh2*, *Cdh11*, *Jag1*, and *Pcdh7*) could be critical to mesoderm development during early embryo development and require further in‐depth investigation.

### 3.6 PCDH7 ^+^ as a specific marker for isolation of mesoderm lineage cells

Out of the 50 mesoderm‐related candidate genes, the membrane surface proteins are of particular interest for further analysis and potential biomedical applications. One such gene *Pcdh7*, as one of the top two most confident and novel DEGs resulting from our stringent screening conditions, was used as an example. According to the MGI review (MGI:1860487), the phenotypes caused by the mutations/alleles of *Pcdh7* gene had strong connections to the hematopoietic and bone marrow system.^65^ More, Behrens et al reported that in a transgenic knockout mouse model, *Pcdh7* was upregulated and affected hematopoietic cells, especially granulocytic and monocytic lineages.^66^ However, the exact relationship between *Pcdh7* function and blood formation has yet to be experimentally verified. *Pcdh7* was shown to be expressed in E11‐E16 fetal liver^67^ which is the main hematopoietic organ during embryonic development,^68^ and fetal liver hematopoiesis peaks at E13.5. Therefore, capturing PCDH7^+^ cells from an E13.5 fetal liver to study hematopoietic differentiation can elucidate whether these PCDH7^+^ cells function in determining mesoderm fate. The fetal liver mononuclear cells (FL‐MNCs) are enriched with hematopoietic stem cells (HSCs), which could be obtained by Ficoll separation method, and can be used for hematopoietic stem cell expansion in vitro^69^ and other mechanistic studies.

**FIGURE 5:**
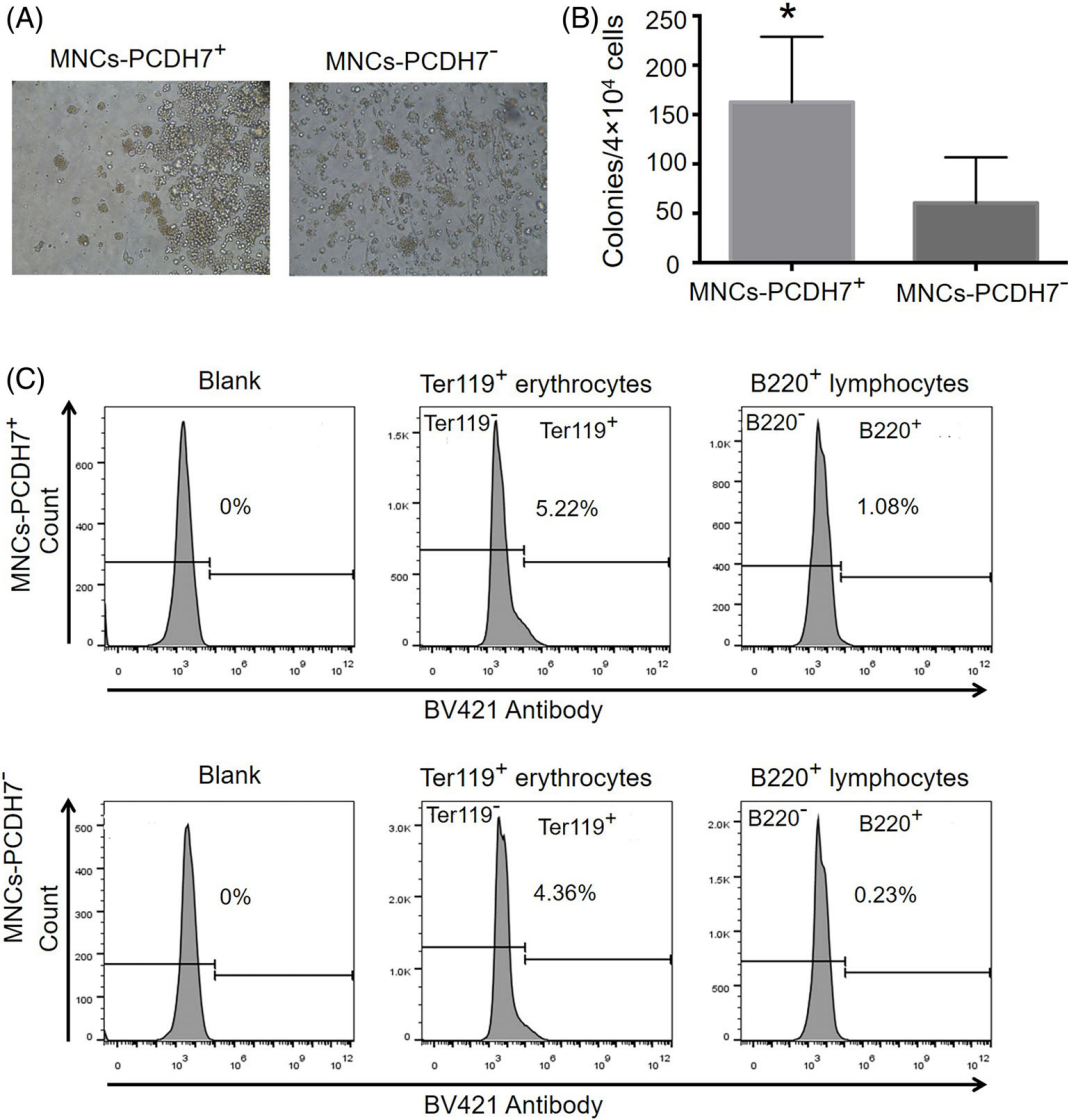
Hematopoietic capacity of PCDH7+ cells from E13.5 fetal liver in vitro. (A) The morphology of cells differentiated from FL‐MNCs at Day 7. (B) The number of CFUs after 7 days of FL‐MNCs differentiation (×200) (*n* = 4 per group). (C) Ter119^+^ erythrocytes and B220^+^ lymphocytes cells were detected using flow cytometry. Data are shown as mean ± SD. *p < 0.05

The effect of *Pcdh7* on the formation of the blood system post mesoderm development process was analyzed by a CFUs assay. CFUs are clonal and reflect the progeny of a single stem/progenitor cell,^70^ that are downstream cells of the embryonic mesoderm. They include erythroid, granulocyte, lymphocyte, macrophage progenitor cells, and terminal blood cells. FL‐MNCs were isolated and the hematopoietic capacity of PCDH7^+^ MNCs evaluated by CFUs experiment in vitro (Figure 5A) showed that the MNCs‐PCDH7^+^ cells formed 2.7 times more colonies than MNCs‐PCDH7^−^ cells (163 ± 66 vs.61 ± 46), indicating the strong hematogenesis ability by PCDH7^+^ cells.

Next, flow cytometry was used to detect the Ter119^+^ cells to analyze the clone formation of erythrocytes, and the B220^+^ cells to analyze the formation of terminal lymph cells. The results showed that MNCs‐PCDH7^+^ fraction could be differentiated into 1.2 times more Ter119^+^ erythroid cells and 4.7 times more B220^+^ lymphocytes than MNCs‐PCDH7^−^ (Figure 5B). This implies that PCDH7 could be a potential marker gene to screen and trace the mesoderm‐specific cells to study their ability to differentiate into downstream cells and terminal cells.

PCDH7 as a potential marker could be used to sort out mesoderm‐derived cell populations with strong hematopoietic ability from E13.5 fetal liver. This is consistent with the fetal liver's ability of hosting hematogenesis progenitor and terminal differentiated cells in that such small population can be enriched using this specific marker. This further proves the robustness and great application potential of using this system to screen mesoderm genes.

## 4 DISCUSSION

Mesoderm is formed during mammalian gastrulation which is a dramatically biological progression. Loss of mesoderm or abnormal development during gastrulation can cause tissue and organ diseases such as myodystrophy, muscular dystrophy, blood disorder, and osteoporosis. Therefore, the study of molecular features of mesoderm and identification of mesoderm‐related genes are necessary for understanding mesoderm development and treatment for mesoderm‐associated diseases.

On the basis of the classic mesoderm morphological structure features of the late streak embryo, samples of the three mesoderm layers were precisely captured by LCM. The origin of samples captured was reliable. Three analytical pipelines were combined and overlapped to screen the candidate mesoderm‐specific genes. GO enrichment analysis, showed that the DEG set has a tendency toward mesoderm development (Table S2). qRT‐PCR, IHC, and flow cytometry based on E7.5 embryos and/or ESCs‐EBs differentiation were used to verify candidate mesoderm‐specific genes expression at RNA, protein, and cellular function levels. Here, an efficient, simple, reliable, and comprehensive system is established to identify the candidate genes at the RNA, protein, and cellular function levels. The results from the verification are consistent with another study,^26^ and it also appears to be more reliable and robust than using only one method for verification. This systematic approach can be extended to screen and validate candidate genes, important in various cellular systems.

Mesoderm cells depended on precise temporal and spatial gene expression regulation to direct proper differentiation. Currently, more marker genes are needed for tracing and purifying the mesoderm cells that express these early regulatory programs. In our study, sections representing mesoderm cells that have the potential for hematopoietic lineages were used for precise cell capture and for transcription analysis of DEGs. Out of the candidate DEGs, a membrane‐associated gene *Pcdh7* with hematopoietic‐relevant functions could be used to trace cells with hematopoietic lineage potential at later stages and has added confidence in the performance of our system.

In conclusion, mesoderm‐related candidate genes obtained from spatial transcriptomic profiling with high confidence have been screened. A simple, efficient, and comprehensive system was described to confirm the candidate genes at the RNA, protein, and cellular function levels. This system can be used to study the fate of subgroups of mesoderm cells during embryo development and can also be extended to studies in other cellular systems.

## AUTHOR CONTRIBUTIONS

Guanghui Liu: experimental design, literature search, performed experiments, data collection, data processing and analysis, drafted the paper. Guanheng Yang: performed experiments, and data collection and processing. Guijun Zhao: data processing and data analysis. Chuanliang Guo: data analysis and revision of the paper. Yitao Zeng: concept, design, and critical review. Yan Xue: experimental design, supervision, and critical review. Fanyi Zeng: concept, experimental design, data analysis, supervision, critical review, and drafted and revised the paper.

## CONFLICT OF INTEREST

The authors declare no conflict of interest.

## Supporting information


**Figure S1** Normalized count of *Cdh2*, *Cdh11*, *Jag1*, and *Pcdh7* in three germ layers samples. Each dot represents an LCM sample of three germ layers. ECT, ectoderm; END, endoderm; MES, mesoderm.

**Table S1** Mesoderm‐specific DEGs (genes differentially expressed higher in mesoderm than ectoderm and endoderm) at late streak stage.

**Table S2** The top 20 significantly (*p*‐value <0.001) enriched GO functions in mesoderm‐specific DEGs from this study.

**Table S3** The top 10 significantly (*p*‐value <0.001) enriched GO functions in mesoderm DEGs from this study.

**Table S4** Top lists of output network molecules, and diseases and functions from IPA analysis for the 50 mesoderm DEGs.

## Data Availability

All related data not included in the manuscript will be available upon request.
