# Supplementary figures and images for: Spatial transcriptomic profiling to identify mesoderm progenitors with precision genomic screening and functional confirmation
Source: Cell Prolif. 2022 Jul 30;55(10):e13298. doi: 10.1111/cpr.13298 (PMC9528766; doi:10.1111/cpr.13298)

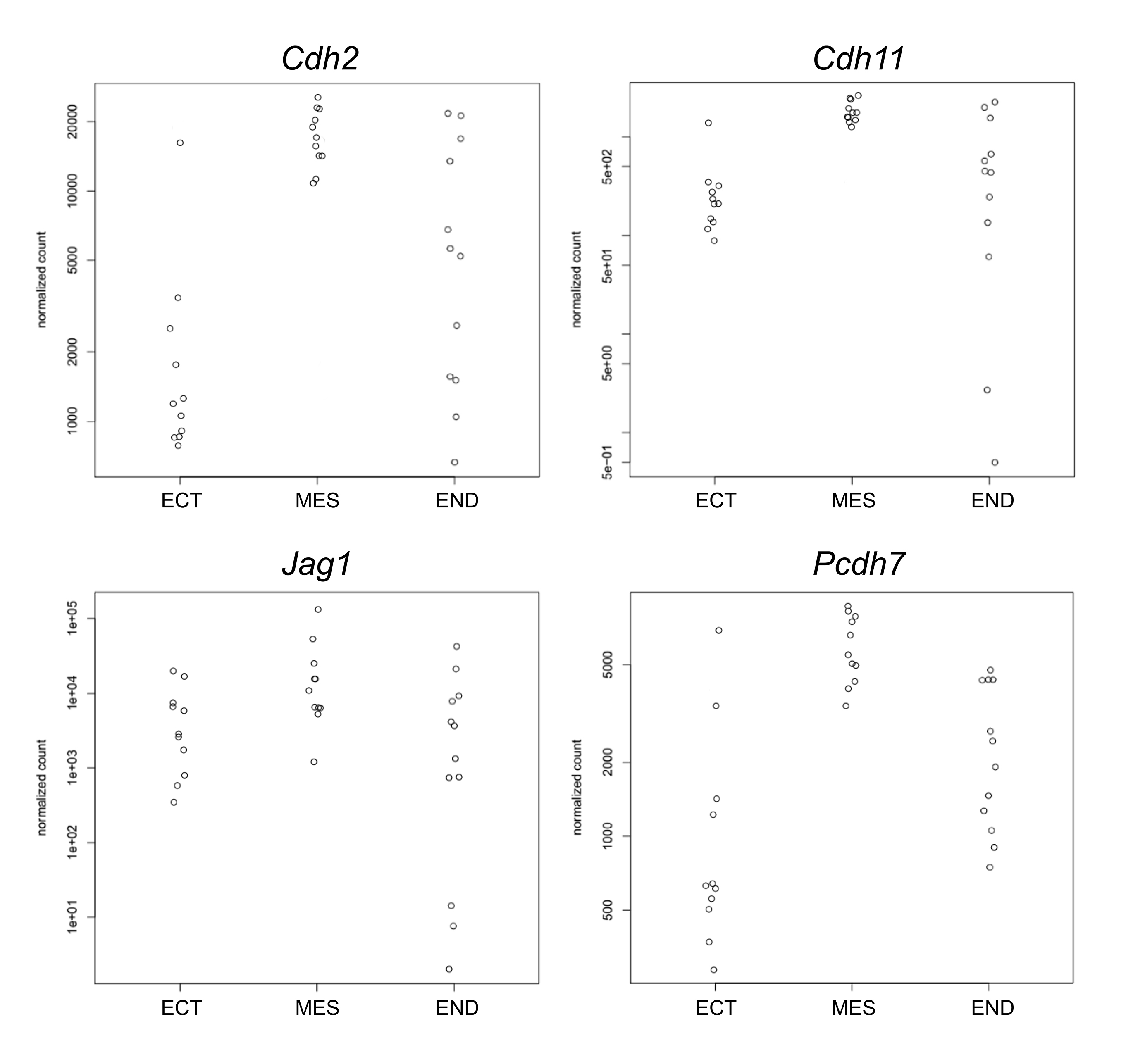

Supplement: Supplementary file 1 — Figure S1 Normalized count of Cdh2, Cdh11, Jag1, and Pcdh7 in three germ layers samples. Each dot represents an LCM sample of three germ layers. ECT, ectoderm; END, endoderm; MES, mesoderm. [file CPR-55-e13298-s004.jpg]
